# American Medical Association

**Published:** 1882-05

**Authors:** William B. Atkinson

**Affiliations:** Permanent Secretary


### ARTICLE IX.

AMERICAN MEDICAL ASSOCIATION, PHILADELPHIA, NO. 1400 PINE ST. The Thirty-third Annual Session will be held in St. Paul, Minn., on Tuesday, Wednesday, Thursday, and Friday, June 6, 7, 8, 9, 1882, commencing on Tuesday, at 11 A. M.

"The delegates shall receive their appointment from permanently organized State Medical Societies, and such county and district medical societies as are recognized by *representation in their respective State Societies*, and from the Medical Department of the Army and Navy, and the Marine Hospital Service of the United States."

"Each State, County, and District Medical Society, entitled to representation shall have the privilege of sending to the association one delegate for every ten of its regular resident members, one for every additional fraction of more than half that number; *provided*, however, that the number of delegates for any particular State, territory, county, city, or town, shall not exceed the ratio of one in ten of the resident physicians who have signed the Code of Ethics of the Association."

Secretaries of medical societies as above designated are earnestly requested to forward, *at once*, lists of their delegates.

SECTIONS.

"The chairmen of the several sections shall prepare and read in the general sessions of the association, papers on the advances and discoveries of the past year in the branches of science included in their respective sections. * * *"—By-Laws, Art. II., Sec. 4.

*Practice of Medicine, Materia Medica, and Physiology*—Dr. J. A. Octerloney, Louisville, Ky., chairman; Dr. D. J. Roberts, Nashville, Tenn., secretary.

*Obstetrics and Diseases of Women and Children*—Dr. H. O. Marcy, Boston, Mass., chairman; Dr. C. V. Mottram, Lawrence, Kan., secretary.

*Surgery and Anatomy*—Dr. ——— ———, chairman; Dr. W. A. Byrd, Quincy, Ill., secretary.

*State Medicine*—Dr. A. L. Gihon, U. S. Navy, chairman; Dr. J. H. Sears, Waco, Texas, secretary.

*Opthalmology, Otology, and Laryngology*—Dr. ——— ———, chairman; Dr. J. Solis Cohen, Philadelphia, secretary.

*Diseases of Children*—Dr. S. C. Busey, Washington, D. C., chairman; Dr. William Lee, Baltimore, Md., secretary.

*Dentistry*—Dr. D. H. Goodwillie, New York City, chairman; Dr. T. W. Brophy, Illinois, secretary.

A member desiring to read a paper before any section should forward the paper, or its *title* and *length* (not to exceed twenty minutes in reading), to the chairman of the Committee of Arrangements at least one month before the meeting.—By-Laws.

*Committee of Arrangements*—Dr. A. J. Stone, St. Paul, Minn., chairman.

*Amendments to the By-Laws*—Offered by Dr. D. H. Goodwillie: Art. II., Sec. 8. Permanent members. Strike out the words "but without the right of voting."

Offered by Dr. J. H. Packard: Regulation II., part I., to read "as permanent members or members by application."

Regulation VI, line 4. Strike out 5 and insert 10. Second paragraph, lines 4 and 5, strike out all after "publication" to and including "association," and insert "publication."

Regulation IX. Add new paragraph: "Members by application shall consist of such members of State and county societies, in good standing, as shall make application in writing for admission. They shall simply have the right to receive the JOURNAL on the same terms as other members."

Regulation IV, paragraph 6. Strike out all from "see," in line 7, to "and" in line 9.

Regulation V, paragraph 3. After "published" insert "in such manner as the association may direct."

WILLIAM B. ATKINSON, M. D.,
*Permanent Secretary.*

